# Whole-Systems Research in Integrative Inpatient Treatment

**DOI:** 10.1155/2013/962729

**Published:** 2013-06-24

**Authors:** Thomas Ostermann, Andre-Michael Beer, Vassya Bankova, Andreas Michalsen

**Affiliations:** ^1^Institute for Integrative Medicine, University of Witten/Herdecke, 58313 Herdecke, Germany; ^2^Department of True Naturopathy, Blankenstein Hospital, Im Vogelsang 5-11, 45527 Hattingen, Germany; ^3^Institute of Organic Chemistry, Centre of Phytochemistry, Bulgarian Academy of Sciences, Academy G. Bonchev Street, Block 9, 1113 Sofia, Bulgaria; ^4^Department of Internal and Complementary Medicine, Immanuel Hospital Berlin, 14109 Berlin, Germany; ^5^Institute of Social Medicine, Epidemiology and Health Economics, Charité University Medical Centre Berlin, 10098 Berlin, Germany

In the last years, medicine has seen a shift from a depersonalized towards a patient-centered individualized medicine. This, on the one hand, is based on the achievements in the field of genetics and molecular science fostering an individually designed drug therapy and treatment tailoring [1]. On the other hand, research on patient-practitioner interaction, psychosocial and behavioral conditions of chronic diseases, and a patient-centered treatment approach has revealed that nonpharmacological factors such as empathy might be essential parameters for the outcome of the patient [2]. Moreover, complementary therapies as traditional whole-medical systems (Ayurveda, traditional Chinese medicine a.o.) or homeopathy, which according to their definition are individualized, have attained increasing interest for improving the patient care, for example, in the treatment of chronic diseases and pains syndromes. More recently, the combination of conventional medicine, complementary medicine, and patient-centered approaches has been put into the conceptional frame of “integrative medicine” which covers these topics adequately [3]. 

The most common and comprehensive definition up to now has been given by the Consortium of Integrative Medicine in 2005. It describes integrative medicine as “the practice of medicine that reaffirms the importance of the relationship between practitioner and patient, focuses on the whole person, is informed by evidence, and makes use of all appropriate therapeutic approaches, healthcare professionals, and disciplines to achieve optimal health and healing” [4]. It therefore joins the latest scientific advances with the profound insights of traditional healing systems and individualized patient care in complementary medicine to regain and preserve health and to enhance self-efficacy and patient's own capacities to recover from illness and to maintain health.

In the last decades, several hospitals have adopted this concept of integrative medicine for the treatment of chronic and acute states of illnesses in an inpatient treatment. For instance, Sendelbach et al. (2003) describe the development of an inpatient integrative therapies program in a cardiovascular tertiary care center [5]. Ernst and Ferrer (2009) also reflect on the implementation of a 7-year integrative hospital program in a cardiac hospital center from the viewpoint of the nursing staff [6]. 

In almost all cases, these hospitals have integrated one or more complementary disciplines as naturopathy, anthroposophical medicine, homeopathy, or traditional Chinese medicine. While, in the USA integrative medicine has emerged towards a marker for innovative quality patient care within the last two decades, other countries have a longer tradition in this field [7]. In Germany, for instance, the amendment to the German Drug Law in 1976 has recognized anthroposophic medicine, homeopathy, and phytotherapy as “Specific Therapeutic Systems” [8]. As a consequence, inpatient treatment opportunities for integrative medicine increased, and specialized hospitals and hospital departments started to evaluate their programs also with respect to comparative cost scenarios [9] which are nowadays still seen as a major goal [10]. Thus, several approaches have already entered the platform of in-patient care in integrative medicine. A current study of Kligler et al. (2011) found a decrease in the use of medications resulting in substantial cost savings in the care of oncology patients treated with an integrative in-patient approach including yoga therapy, holistic nursing, and healing environment [11]. Unfortunately, due to heterogeneity of the approaches and evaluations, the meta-analyses and systematic reviews of such evaluations in most cases suffer from a lack of comparability of outcome parameters within these evaluations so far.

Of note, whole-systems evaluation of integrative in-patient treatment might also benefit from a systematic analysis of the patient characteristics of clinical pathways and flow processes between in- and outpatient treatments, which has been described [12].

Finally, concepts of combining education, teaching, and in-patient integrative treatment can be seen as an important milestone for the development of integrative medicine. If only students are able to discover and get some first insight into the potential different perspectives of medicine and healing, they might be able to argue open-mindedly about the best fitting therapeutic strategy for the individual patient to regain his optimal health [13].

Despite these current achievements, evaluations of the approach of integrative medicine as a whole system and its interactions with other services of patient care have only been seen marginally. Thus, the cutting edge for the future development of integrative medicine now is to close the “evidence gap” [4] and to summarize and communicate these findings of research to the public, the stakeholders, and policy makers of healthcare authorities [14]. 

This special issue tries to step into this direction by bringing together evidence from different perspectives. Apart from whole-systems evaluations of hospital programs, cost studies, and findings on educational aspects of integrative medicine, this issue also covers historical aspects of the development of integrative medicine and presents the meta-analyses of integrative approaches. Hopefully, science-driven implementation of integrative medicine into hospitals and patient care can improve the 21st century medicine.

*Thomas Ostermann* *Thomas Ostermann* *Andre-Michael Beer* *Andre-Michael Beer* *Vassya Bankova* *Vassya Bankova* *Andreas Michalsen* *Andreas Michalsen*
